# Differential Nasal Expression of Heat Shock Proteins 27 and 70 by Aerobic Exercise: A Preliminary Study

**DOI:** 10.7150/ijms.39631

**Published:** 2020-02-24

**Authors:** Hyun Jin Min, Sung Jin Min, Hyun Kang, Kyung Soo Kim

**Affiliations:** 1 Department of Otorhinolaryngology-Head and Neck Surgery, College of Medicine, Chung- Ang University Hospital, Seoul, Republic of Korea.; 2 Department of Anesthesiology and Pain Medicine, College of Medicine, Chung- Ang University Hospital, Seoul, Republic of Korea.

**Keywords:** Hsp70, Hsp27, nasal mucosa, exercise, athletes

## Abstract

**Purpose:** Exercise modifies airway immune responses and susceptibility to infection. We investigated the effects of exercise on two HSPs (heat shock proteins), quantifying expression levels in nasal mucosa of both professional competitive athletes and non-athletes for comparison.

**Method:** We used western blot technique to determine expression levels of HSPs in primary human nasal epithelial cells (HNECs). Nasal lavage (NAL) fluids were collected from 12 male professional volley ball players and 6 healthy males pre-submaximal exercise (running for 30 min at 70-80% of maximal heart rate) and post-submaximal exercise. Expression levels of HSP27, HSP70, Interleukin (IL)-8, and Tumor necrosis factor (TNF)-α in NAL fluids were quantified by enzyme-linked immunosorbent assay (ELISA), and difference of the level between pre-submaximal exercise and post-submaximal exercise was statistically analyzed. Antibacterial assay using *Staphylococcus aureus* was performed to assess the immunological role of HSPs in NAL fluids.

**Results:**. In non-athlete controls, HSP27, HSP70, and IL-8 were unchanged after exercise. In the professional athletes, HSP70 expression was declined significantly (*p*<0.05), but HSP27 was not significantly changed. IL-8 and TNF-α did not show significant difference, either. By antibacterial assay, it was found that the number of active bacterial populations were influenced by the presence or absence of HSP27 and HSP70 in NAL fluids.

**Conclusion:** HSP27 and HSP70 were present in NAL fluids of enrolled subjects, and the effect of exercise on the level HSPs was different between professional athletic competitors and non-athletes. As the number of active bacterial population was influenced by the presence or absence of nasal HSP27 and HSP70, we suggest that HSP27 and HSP70 may play immunological function in NAL fluids.

## Introduction

Heat shock proteins (HSPs) are molecular chaperones that govern proper protein folding, refolding of misfolded proteins, and degradation of irrecoverable proteins 1,2. HSP families are categorized by molecular weight (i.e. small HSPs, HSP60, HSP70, and HSP90). These proteins are detectable under physiological conditions but may be induced by various stresses, such as heat shock, oxidative stress, osmotic pressure instability, and ischemic injury 1. In addition to their initially identified roles as molecular chaperones, HSPs also seem to function as damage-associated molecular pattern (DAMP) molecules in various organs 3-5. Of the various HSP isoforms, HSP27 and HSP70 are currently considered biomarkers in the monitoring of exercise training and adaptive mechanisms 6,7.

Exercise influences on immune system by triggering critical signaling pathways and upregulating inflammatory mediators 7. There is evidence that high-intensity treadmill exercise increases HSP expression levels in cardiac muscles 8 and that acute resistance exercise induces phosphorylate Sestrin 2 expression in human skeletal muscle 9. In football players, training boosts the levels of proteins related to mitochondrial biogenesis and improves the antioxidant capabilities of mitochondria in peripheral blood mononuclear cells 10. Likewise, high-intensity exercise readily raises leukocyte counts and augments other analytes in human blood, including myeloperoxidase (MPO), polymorphonuclear leukocyte (PMN) elastase, cortisol, creatine kinase activity, myoglobin, interleukin (IL)-6, IL-10, and high-sensitivity C-reactive protein (hs-CRP) 11.

In airways, the mechanical stress of labored breathing during exercise may damage epithelial cells and promote the release of mediators that induce inflammatory reactions 12,13. It has been reported that unlike healthy controls, exercise prompted a slight increase in HSPs of exhaled breath condensates produced by athletes 14. Furthermore, monocyte chemoattractant protein 1 (MCP-1) and IL-16 in exhaled breath condensates of children faced with positive exercise challenges (vs unchallenged counterparts) have proven to be significantly higher 15; and in sputum of elite athletes (vs controls), measured baseline levels of uric acid, high-mobility group box-1, and IL-8 mRNA have been significantly higher 12.

To our knowledge, expression levels of HSP27 and HSP70 (both viewed as exercise-related biomarkers) have yet to be investigated in the upper airways before and after aerobic exercise. Likewise, a comparison between athletic competitors and non-athletes could not be investigated in the previous studies using the method of nasal lavage or mucosal fluids. This study was conducted to investigate the effects of aerobic exercise on upper airway expression of HSP27 and HSP70, comparing outcomes obtained in professional athletic competitors and in non-athlete controls.

## Materials and Methods

### Subject selection and collection of nasal lavage fluids

This study was approved by the Institutional Review Board of Chung-Ang University College of Medicine (132-009-276), and all those who participated granted informed consent. Six healthy male volunteers who exercised <30 min/week served as control subjects (Table 1), and enrolled subjects were free from injury, smoking, no flu or upper respiratory inflammation had been reported in the preceding 3 months. All were instructed to refrain from alcohol, caffeine, and intense physical activity throughout the experimentation period. On the day of testing, each performed aerobic exercise (running for 30 min, non-stop, 20 ºC, 60% of humidity level) at submaximal level (70-80% of maximal heart rate). Before and after exercising, NAL fluids were collected. Twelve professional male volleyball players who exercised daily (at least 2 h) volunteered as test subjects (Table 1). Participants performed 30 minutes run on the treadmill at 70-80% from the max HR in a climatic condition of 20 ºC and 60% humidity, respectively. Exercise testing and NAL fluid collection was performed at the same time of the day in each group.

### Cell culture

Human nasal epithelial cells (HNECs) were purchased (PromoCell; Heidelberg, Germany), and cultured (37 °C, 5% CO_2_, fully humidified) in airway epithelial cell growth medium. Subcultures were established, according to manufacturer's instructions 16. The cells were incubated (45 min, 39.5 °C) and harvested 1 h after heat shock for our purposes.

### Concentration of supernatants

Culture supernatants, collected to evaluate HSP expression levels, were concentrated in accord with our previously described protocol 17. Briefly, same numbers of HNECs were seeded in 6-well plates and heat shock was induced. Entire amounts of culture supernatants were collected and concentrated (Centrifugal Filter Unit; MilliporeSigma, Burlington, MA, USA). Concentrated samples were then mixed (x 5) with sample buffer, and western blot was performed.

### Western blot technique

Concentrated culture supernatant samples were separated on 12% SDS-PAGE gel, transferring proteins to a nitrocellulose membrane. Primary antibodies against HSP27, HSP70, HSP90 (Abcam, Cambridge, UK) and HRP-labeled goat anti-mouse Ig (Jackson Laboratory, Bar Harbor, ME, USA) secondary antibodies were applied, using ECL reagent (Amersham Biosciences, Piscataway, NJ, USA) for detection.

### Nasal lavage fluid collection

Nasal lavage fluid was sampled from six healthy male non-athletes and twelve professional male volleyball players, adhering to a previously detailed protocol. Briefly, 5 ml of normal saline was instilled into the nasal cavity via syringe 18. As subjects were supine position, some amount of instilled normal saline was got out of the posterior nasal airway, and some was entered to middle meatus, leaving 2.5 ml of nasal secretions by suction prior to intranasal manipulation. NAL fluid collection was performed following our previous protocols 19-21. The secretions were immediately centrifuged to remove mucus and stored (-70°C) until needed.

### HSP27, HSP70, IL-8, and TNF-α determinations

Expression levels of HSP27, HSP70, and IL-8 in nasal lavage fluids were quantified using a commercially available enzyme-linked immunosorbent assay (ELISA) as directed (R&D Systems, Minneapolis, MN, USA).

### Bacterial preparation

*Staphylococcus aureus* (1 x 10^8^ CFU/mL) were suspended for collection by centrifugation. After washing three times and re-suspension in sterile saline (1 mL), a 10-μL aliquot was applied to a 96-well plate (1 x 10^6^ cells) for subsequent experiments.

### Precipitation of HSP27 and HSP70 from lavage fluids and antibacterial activity assay

Nasal lavage fluid (1 mL) obtained in steady status was used to evaluate functional changes in professional volleyball players. To preclear the specimens, they were incubated with Protein G Sepharose beads (MilliporeSigma). The precleared samples were then incubated (overnight) with anti-HSP27 and anti-HSP70 antibodies and ultimately centrifuged to remove these proteins, using the supernatant for anti-bacterial assay. A portion (100 μL) of the residual nasal lavage fluid was added to culture wells (1 x 10^6^ cells) containing *S. aureus* and sterile saline (10 μL). The cells were incubated (18 h, 37°C) in a shaking incubator and harvested. Harvested fluids containing *S. aureus* were serially diluted, and the colonies formed on brain heart infusion agar plates after incubation (8 h) were counted (pour plate method). HSP27 and HSP70 proteins were precipitated using anti-HSP27 and HSP70 antibodies and removed by centrifugation. These precipitated proteins were later confirmed by western blot.

### Statistical analysis

All computations relied on standard software (SPSS v21.0; IBM, Armonk, NY, USA). The Wilcoxon signed rank test was used to compare levels of HSP27 and HSP70 present in nasal lavage fluids pre- and post-submaximal aerobic exercise. Between-group differences in HSP27 and HSP70 were analyzed using a linear mixed-effects model (LMEM), whereby timing (pre- and post- submaximal aerobic exercise) and subject groups (volleyball players vs controls) constituted independent fixed factors, and individual participants represented random effects. Statistical significance was set at *p*<0.05.

## Results

### HSP27 and HSP70 in nasal lavage fluid of non-athletes (pre- and post- submaximal aerobic exercise)

**Figure 1:**
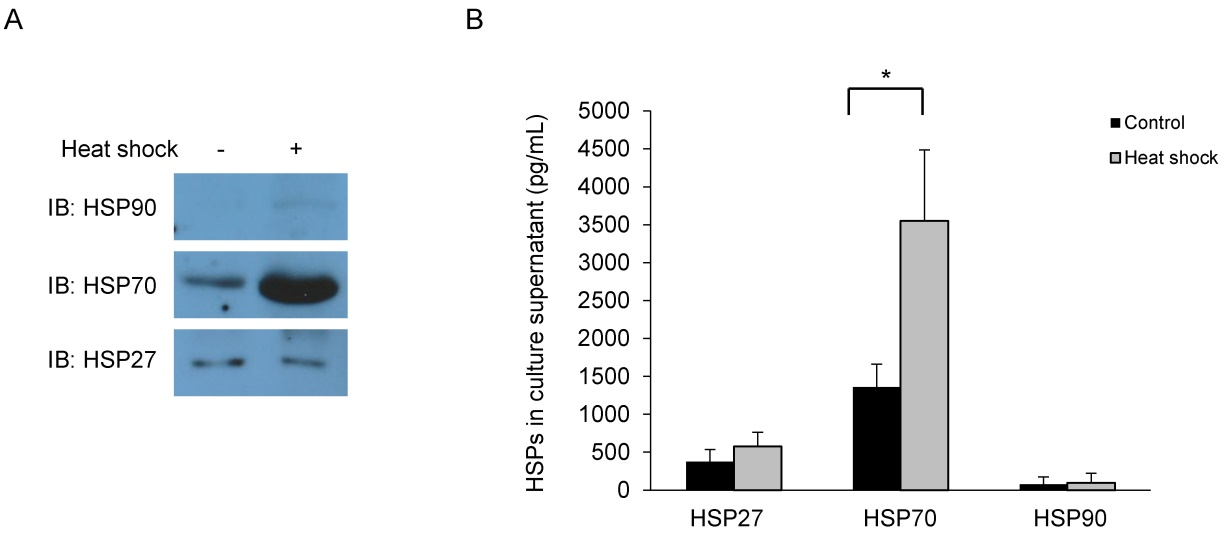
Primary human nasal epithelial cells are harvested 1 h after heat shock for (A) Western blot using antibodies as indicated and (B) supernatant analysis (ELISA). *p<0.05, N=3.

The expression and secretion of HSPs were evaluated by whole cell lysates and culture supernatant of Primary HNECs. Western blotting of whole cell lysates indicated that HSP27 and HSP70 were highly expressed, whereas HSP90 expression was negligible (Fig. 1A). The concentrated culture supernatants were also tested (ELISA), again identifying HSP27 and HSP70. However, HSP90 was undetected, as in the western blot (Fig. 1B). Based on these results, we presumed that upper airway HSP expression was largely confined to HSP27 and HSP70.

**Figure 2:**
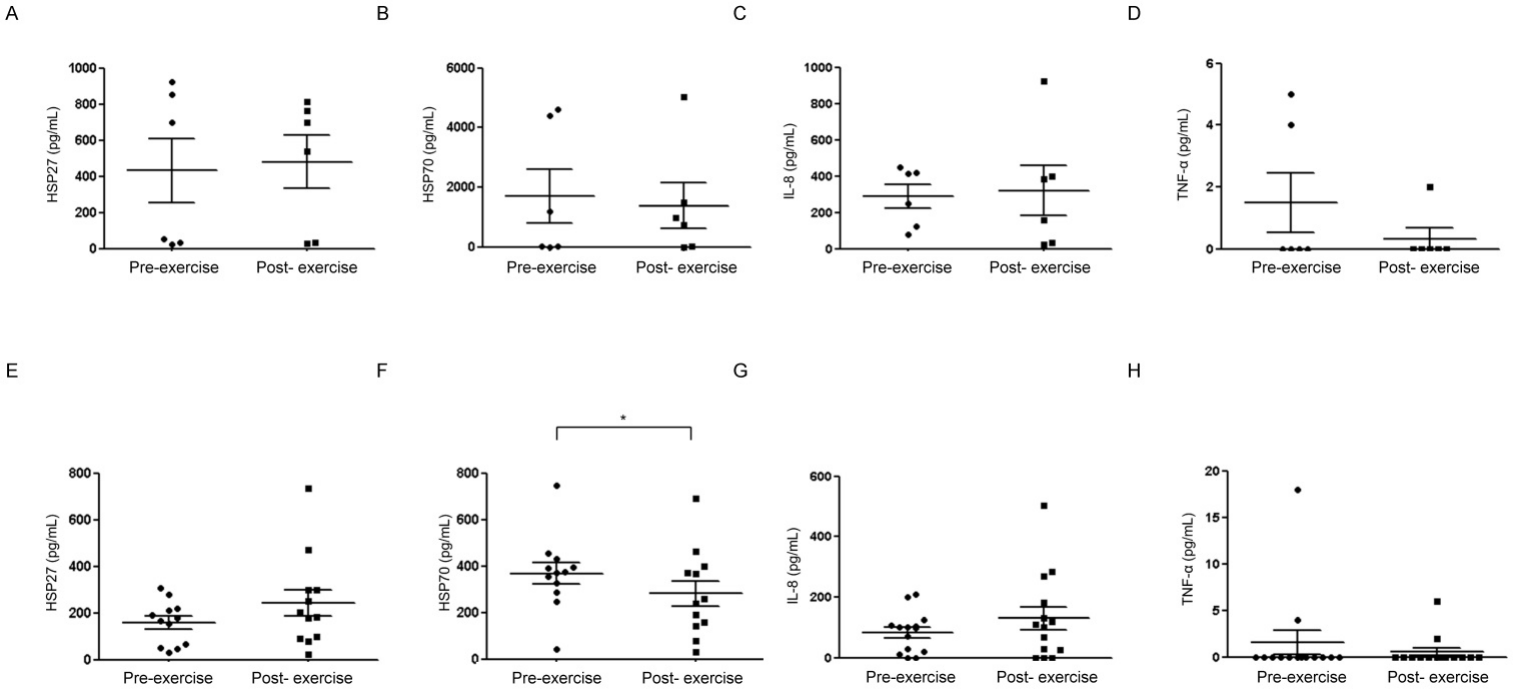
Nasal lavage fluids collected from non-athlete controls (A-D) and competitive athletes (E-H) pre-and post-submaximal aerobic exercise for comparative analysis (ELISA) of HSP27, HSP70, IL-8, and TNF-α. *p<0.05, N=3.

Next, ELISA assay was performed using NAL fluids from non-athletes (controls), which were as follows: HSP27, 431.68 ± 436.94 pg/mL (pre-submaximal exercise) and 479.75 ± 360.02 pg/mL (post- submaximal exercise); HSP70, 1701.61 ± 2216.63 pg/mL (pre-submaximal exercise) and 1370.82 ± 1874.20 pg/mL (post-submaximal exercise). Levels present before and after aerobic exercise did not differ significantly (Fig. 2A, 2B). In measuring IL-8 (which was detected and is important in upper airway inflammatory pathways) 20, levels present before (288.94 ± 161.37 pg/mL) and after (319.70 ± 338.49 pg/mL) exercise were similar (Fig. 2C). Another inflammatory mediator, TNF-α, was identified in the fluid of three subjects only and showed no significant differences (Fig. 2D) 19.

### HSP27 and HSP70 in nasal lavage fluid of professional competitive volleyball players (pre- and post-submaximal aerobic exercise)

HSP27 present pre-exercise (158.37 ± 92.08 pg/mL) increased somewhat post-exercise (242.23 ± 197.57 pg/mL), and there was no significant different in HP27 at pre to post exercise between professional volley ball players and the control group (Fig. 2E). On the other hand, HSP70 present before exercise (368.50 ± 161.26 pg/mL) declined significantly after exercise (282.24 ± 185.71 pg/mL) (p<0.05) (Fig. 2F). In measuring IL-8, levels present before (83.92 ± 67.58 pg/mL) and after (130.14 ± 141.76 pg/mL) exercise were similar (Fig. 2G). Another inflammatory mediator, TNF-α, was identified in the fluid of four subjects only and showed no significant differences (Fig. 2H).

### Comparison of HSP27 and HSP70 levels pre- and post-submaximal aerobic exercise in professional competitive volleyball players and non-athletes (controls)

**Figure 3:**
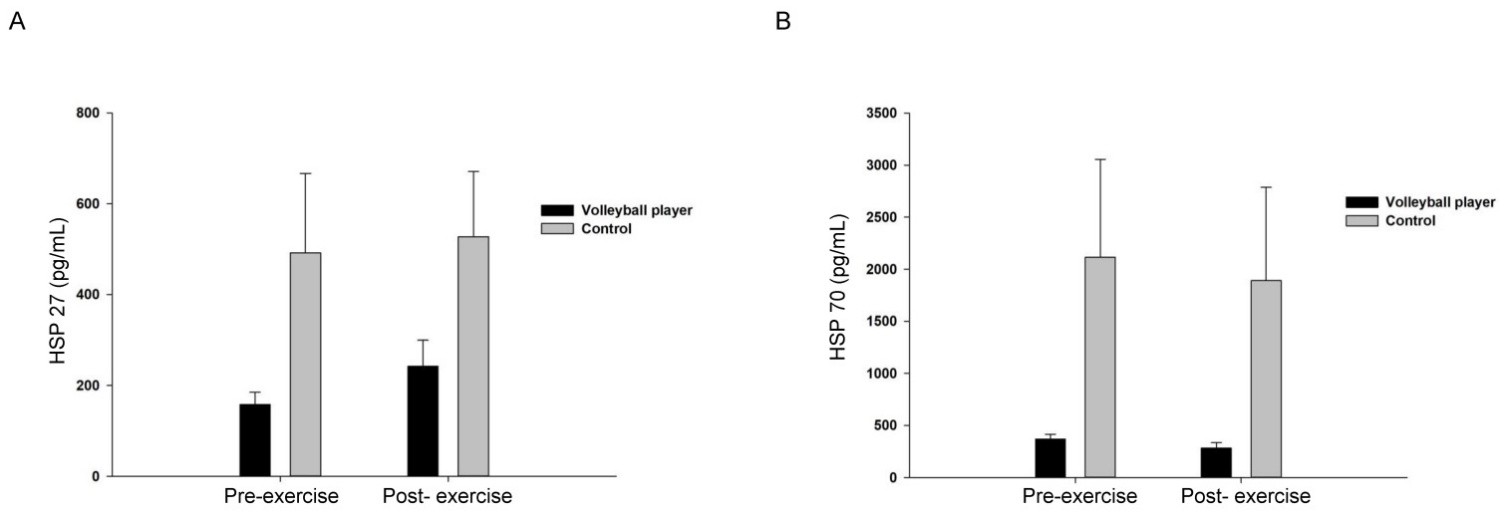
Analysis of between-group differences in HSP27 (A) and HSP70 (B) using a linear mixed-effects model to account for timing (pre- and post- submaximal aerobic exercise) and subject groups (volleyball players vs controls).

Next, evaluation between-group differences using LMEM was performed. With respect to HSP27, LMEM revealed a significant difference between professional volleyball players and non-athletes (F [1, 32.994] = 8.110; MD [mean difference]: -255.65 [-438.30 to -73.01]; *p* = 0.008) but no significant difference between pre- and post-submaximal aerobic exercise. Similarly, LMEM confirmed a significant difference of HSP70 between professional volleyball players and non-athletes (F [1, 32.175] = 8.963, MD: -1191.62 [-2002.21 to -381.02];* p* = 0.005), but no significant difference between pre- and post-submaximal aerobic exercise (Fig. 3).

### Nasal lavage fluids of professional competitive volleyball players show increased anti-bacterial activity after aerobic exercise

**Figure 4:**
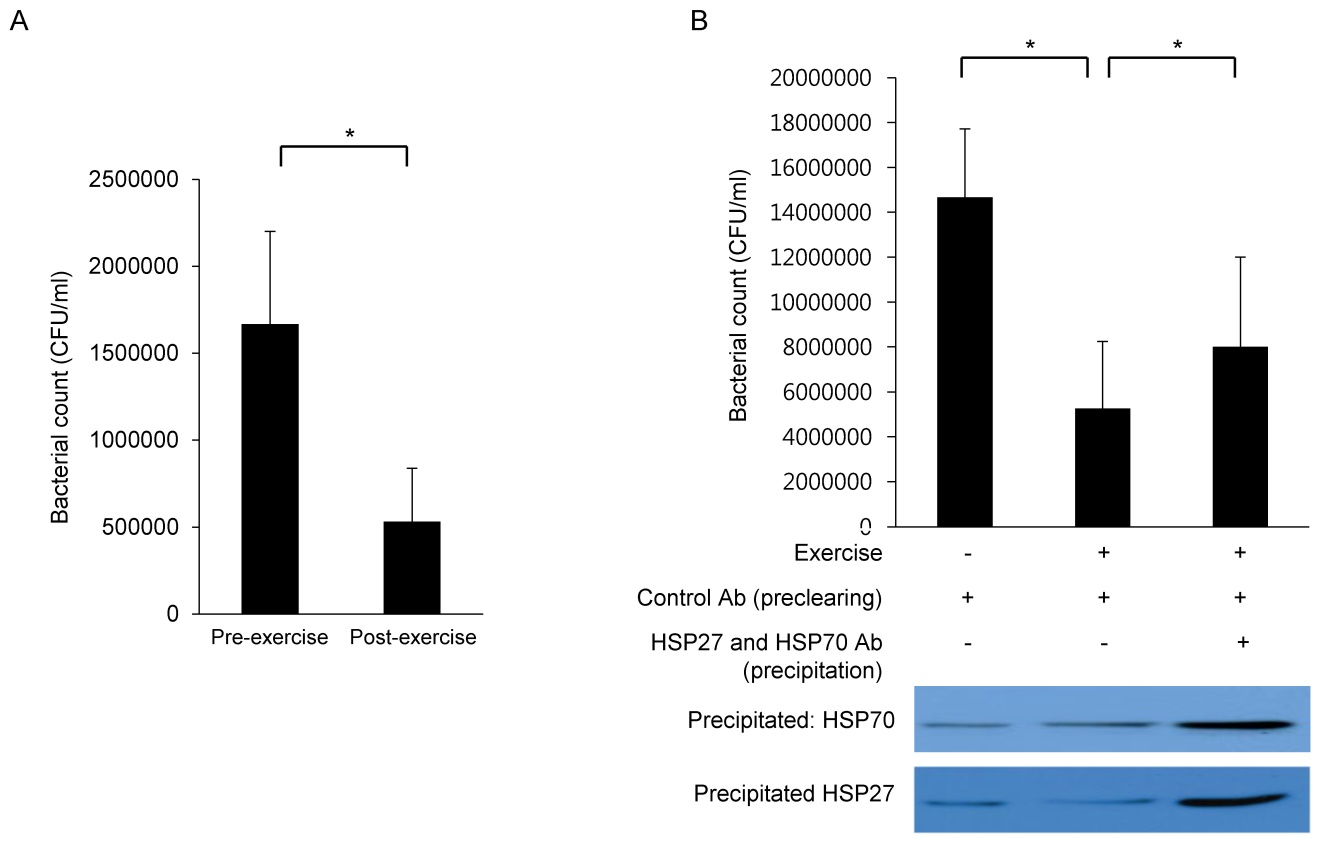
The number of bacterial population is reduced in nasal lavage fluids collected pre-and post-submaximal aerobic exercise (A). Reduced number of bacterial population is recovered (B, upper panel) when HSP27 and HSP70 were precipitated and removed from nasal lavage fluids (B, lower panel). (+ stands for presence of exercise or indicated antibody, and - stands for absence of exercise or indicated antibody). *p<0.05, N=3.

Finally, the functional roles of HSP27 and HSP70 were evaluated by antibacterial activity assay. Once *Staphylococcus aureus* (*S. aureus*) was incubated with NAL fluids collected after exercise, bacterial populations were reduced compared to NAL fluids collected before exercise (Fig. 4A), suggesting that nasal anti-bacterial activity is heightened after exercise. To further determine the anti-bacterial potential of HSP27 and HSP70, these proteins (antigen-antibody complexes) were precipitated from the lavage fluid. Precipitated antibody-bound HSP27 and HSP70 were later confirmed by western blot (Fig. 4B, lower panel). Upon removal of HSP27 and HSP70, the initially observed augmentation of anti-bacterial activity in post-exercise NAL fluids was abolished (Fig. 4B, upper panel). These findings were statistically significant.

## Discussion

In this study, levels of HSP27 and HSP70 were assessed pre- and post-submaximal aerobic exercise in both competitive athletes (professional volleyball players) and non-athlete controls for comparison purposes. The possibility that HSP27 and HSP70 may play immunological role in NAL fluids was also assessed.

Accordingly, it was found that basal levels of HSP27 and HSP70 in nasal lavage fluids of professional competitive volleyball players and non-athlete controls differed significantly.

Despite the many subsets of HSPs, HSP27 and HSP70 are currently regarded as novel biomarkers, enabling one to monitor exercise training and adaptive mechanisms 6,7. An upsurge in HSP27 after exercise may concurrently be involved in regulating free radicals and combatting the toxicity of reactive oxygen species (ROS) 22. HSP70 released into extracellular area during exercise contributes to the sensation of fatigue and may signify an immunomodulatory response that culminates in the binding of various receptors 23. This is the first report, as far as we know, that HSP27 and HSP70 are indeed released into nasal lavage fluids, and the levels fluctuate under certain circumstances. Hence, they may well be involved in upper airway immune responses triggered by exercise.

In the professional volleyball players (vs non-athletes), basal levels of HSP27 and HSP70 proved to be comparatively lower. These competitive athletes engage daily in aerobic exercise, regularly invoking stress responses that may influence basal HSP status. It was reported that in endurance sport, change of membrane integrity and migration of inflammatory cells result in higher prevalence of upper respiratory tract infection 24. Likewise, other inflammatory mediators in the upper airway, such as TNF-α and IL-1ra, have shown differential expression in competitive athletes and healthy controls 14. These findings imply that professional athletes (or others) who indulge in prolonged exercise programs may deviate in their immune responses, thus altering defense mechanisms against upper airway infections. In this study, it was found that nasal HSP70 expression declined significantly in professional competitive volleyball players after exercise. As extracellular HSP70 may function as DAMP molecule, our finding suggests that innate immune response induced by exercise could be different from that of non-athletes.

IL-8 is a neutrophilic chemokine that is freely present in nasal lavage fluid and is a known associate of other DAMP molecules in chronic rhinosinusitis 19,20. In this experiment (as in other reports), IL-8 was readily detected in all nasal lavage fluids; and although one particular study has demonstrated that in lower airways, baseline CXCL8 mRNA levels of sputum measured in swimmers exceeded those of controls 12. Furthermore, IL-8 level was unchanged after exercise and bore no relation to levels of HSP27 and HSP70 (also DAMPs). Therefore, we believe that secretion of DAMPs in upper airways is regulated separately during- each stressful condition (such as exercise).

Most notably, it was found that nasal HSP27 and HSP70 levels in response to exercise were different for professional competitive volleyball players and non-athletes. Although there were case reports that exercise changed the level of HSP levels 7, this is the first report that suggests the possibility that response of HSPs after exercise may be different between athletes and non-athletes (Fig. 3).

The final aspect of our investigation was aimed at defining the immunologic functions of HSP27 and HSP70 in nasal lavage fluid sampled before and after exercise. *S. aureus* enterotoxin B (SEB) exposure initiates a rapid induction of HSP25 and HSP72 in intestinal epithelial cells that likely is pivotal in protecting the gut from damaging bacterial infections 25. *S. aureus* is a notorious upper airway pathogen, especially in instances of sinusitis 26. Therefore, *S. aureus* was targeted in this study when evaluating the anti-bacterial activity of nasal lavage fluids. And it was found that lavage samples collected after exercise decreased the colonization of *S. aureus*. This inhibitory effect was also partly nullified by removal of HSP27 and HSP70 from the fluid samples (Fig. 4). Our previous studies have shown that recombinant HSP27 and HSP70 proteins promote *S. aureus* upregulation* in vitro.* However, the present results are somewhat incongruous. Nasal lavage fluids obtained after (vs before) exercise contained higher levels of HSP27 and lower levels of HSP70, perhaps exerting mixed effects on bacterial growth. We feel that anti-bacterial activity may predominate, but the mechanisms involved should be further explored.

The present study has several limitations. First, the number of enrolled subjects is relatively small. This is a prospective study, and there was limitation in recruitment of control subjects. Another issue is that we did not originally check for the presence of allergic diseases, AR in particular and rhinosinusitis. As previously documented, AR or presence of concurrent rhinosinusitis may affect levels of HSPs in nasal lavage fluids 21. Because our study participants were so few, they could not be stratified by AR status for statistical analysis. The results may have differed, had this been possible. A large number of subjects with or without AR should be evaluated in this regard. Also, the duration of exercise could not be enough to trigger definite change in HSP level. And other type of exercise could results in different results, for example, an athlete who practices outdoor sports would likely exhibit very different results as most study have shown that outdoor activity has a great effect on the upper respiratory system. Finally, our functional analysis of HSP27 and HSP70 in nasal lavage fluids relied solely on *S. aureus. S. aureus* is one of normal flora, and other bacterial strains should be considered as well to affirm the anti-bacterial effects of HSP27 and HSP70 in upper airways pre- and post-submaximal aerobic exercise.

### Perspective

The effect of aerobic exercise on innate immunity in upper airway, especially regarding DAMPs, have not been much evaluated. This is the first report that the levels of nasal HSP27 and HSP70, which function similar to DAMPs in innate immune response, remained unchanged pre-and post-submaximal aerobic exercise in non-athletes, and nasal HSP70 expression declined significantly in professional competitive volleyball players after exercise. Our data suggest that the differential nasal expression of HSP27 and HSP70 observed after aerobic exercise in competitive athletes and non-athletes may confer differing susceptibilities to infection, and following studies may further support our findings.

## Figures and Tables

**Table 1:** Anthropometric measurements of non-athlete controls and professional volleyball players

Variables	Mean±SD Non-Athletes	Mean±SD Volleyball Players
Age, years	32.16±3.92	29.16±4.56
Height, cm	176±5.05	191±8.57
Body weight, kg	72.83±6.52	84.83±9.02
Body mass index, kg/m^2^	23.53±2.18	23.13±0.97

SD: standard deviation.
